# Supplementary material for: COVID-19 Vaccination in People Living with HIV (PLWH) in China: A Cross Sectional Study of Vaccine Hesitancy, Safety, and Immunogenicity
Source: Vaccines (Basel). 2021 Dec 9;9(12):1458. doi: 10.3390/vaccines9121458 (PMC8706383; doi:10.3390/vaccines9121458)
Supplement: Supplementary file 1 [file vaccines-09-01458-s001.zip › vaccines-1486750-supplementary.pdf]

Table S1 Full Questionnaire of Vaccination status and attitudes to COVID-19 vaccine

Section A

DEMOGRAPHICS, HIV CHATACTERISTICS AND HEALTH STATUS

Demographics		
1	Age	______________________years old
2	Gender	[1] Male<br>[2] Female
3	Marital status	[1] Unmarried<br>[2] Married<br>[3] Divorced/widowed
4	Highest education level	[1] High school and below<br>[2] College/undergraduate<br>[3] Postgraduated and above
5	Occupation	[1] Business/service staff<br>[2] Professional and technical personnel<br>[3] Public official<br>[4] Farmer/industrial worker<br>[5] Student<br>[6] Unemployed<br>[7] Others
HIV characteristics		
6	Duration of antiretroviral therapy	______________________years
7	antiretroviral therapy regimen	______________________
8	$CD4^+$ T cell counts prior to antiretroviral therapy initiation	______________________cells/μL
9	HIV RNA load to antiretroviral therapy initiation	______________________copies/ml
10	$CD4^+$ T cell counts before 6 months	______________________cells/μL
11	HIV RNA load before 6 months	______________________copies/ml
12	Mode of HIV transmission	[1] MSM<br>[2] Heterosexual sex

		[3] Transfusion<br>[4] Others/unknown
Short Form Health Survey (SF-12)		
14	In general, would you say your health is	[1] Excellent<br>[2] Very good<br>[3] Good<br>[4] Fair<br>[5] Poor
15	The following questions are about activities you might do during a typical day. Does your health now limit you in these activities? If so, how much?<br>a. Moderate activities, such as moving a table, pushing a vacuum cleaner, bowling, or playing golf<br><br>b. Climbing several flights of stairs	 <br><br><br>[1] Yes, limited a lot<br>[2] Yes, limited a little<br>[3] No, not limited at all<br><br>[1] Yes, limited a lot<br>[2] Yes, limited a little<br>[3] No, not limited at all
16	During the past 4 weeks, have you had any of the following problems with your work or other regular daily activities as a result of your physical health?<br>a. Accomplished less than you would like<br><br>b. Were limited in the kind of work or other activities	 <br><br><br>[1] Yes<br>[2] No<br><br>[1] Yes<br>[2] No
17	During the past 4 weeks, have you had any of the following problems with your work or other regular daily activities as a result of any emotional problems (such as feeling depressed or anxious)<br>a. Accomplished less than you would like<br><br>b. Did work or other activities less carefully than usual	 <br><br><br>[1] Yes<br>[2] No<br><br>[1] Yes<br>[2] No
18	During the past 4 weeks, how much did pain interfere with your normal	[1] Not at all<br>[2] A little bit

	work (including both work outside the home and housework)?	[3] Moderately<br>[4] Quite a bit<br>[5] Extremely
19	These questions are about how you feel and how things have been with you during the past 4 weeks. For each question, please give the one answer that comes closest to the way you have been feeling.<br>How much of the time during the past 4 weeks.<br>a. Have you felt calm and peaceful?	 <br><br><br><br><br><br><br>[1] All [2] Most<br>[3] A good bit [4] Some<br>[5] A little [6] None
	b. Did you have a lot of energy?	[1] All [2] Most<br>[3] A good bit [4] Some<br>[5] A little [6] None
	c. Did you have a lot of energy?	[1] All [2] Most<br>[3] A good bit [4] Some<br>[5] A little [6] None
20	During the past 4 weeks, how much of the time has your physical health or emotional problems interfered with your social activities (like visiting friends, relatives, etc.)?	[1] All<br>[2] Most<br>[3] A good bit<br>[4] Some<br>[5] A little<br>[6] None

Section B

Perception of COVID-19 vaccine

1	Have you get vaccinated against COVID-19?	[1] Yes<br>[2] No
2	If your answer is no, what's preventing you from getting vaccinated?	[1] Concerns about side effects and/or poor efficacy<br>[2] Be waiting to be scheduled<br>[3] Contraindications for vaccine<br>[4] No perceived need for vaccination<br>[5] Scheduling conflicts

3	If your answer is yes, do you have any concern after vaccination?	______________________________

Section C (vaccinees only)

Status of vaccination against COVID-19

1	The manufacturer of COVID-19 vaccine	[1] CoronaVac vaccine (Sinovac Life Sciences)<br>[2] BBIBP-CorV vaccine (Beijing Institute of Biological Products)<br>[3] Recombinant protein subunit vaccine (Anhui Zhifei Longcom Biopharmaceutical)<br>[4] Recombinant adenovirus type-5 vectored vaccine (CanSino Biologics)
2	How many doses have you completed? When did you get vaccinated	[1] First dose. Date:________________<br>[2] Second dose. Date:______________<br>[3] Third dose. Date:_______________

Section D (vaccinees only)

Adverse reactions related to COVID-19 vaccine

Local adverse reactions		
1	☐Pain at injection site	[1] Didn't affect or slightly affect physical activity<br>[2] Affected physical activity<br>[3] Affected daily life<br>[4] Led to loss of self-care ability or needed to be in hospital<br>Duration of symptom: ___________________days
2	☐Swelling	[1] 2.5~<5 cm in diameter / 6.25~<25 cm^2 in area and didn't affect or slightly affected physical activity<br>[2] ⩾10 cm in diameter/⩾100 cm^2 in area/got a fester or secondary infection or phlebitis or sterile abscess or wound drainage/seriously affected quality of life<br>[3] ⩾10 cm in diameter/⩾100 cm^2 in area/got a

		fester or secondary infection or phlebitis or sterile abscess or wound drainage/seriously affected quality of life<br>[4] Abscess / exfoliativedermatitis/dermal or deep tissue necrosis<br>Duration of symptom: ____________________days
3	☐Redness	[1] 2.5~<5 cm in diameter/6.25~<25 cm^2 in area and didn't affect or slightly affected physical activity<br>[2] 5~<10 cm in diameter/25~<100 cm^2 in area/affected physical activity<br>[3] ⩾10 cm in diameter/⩾100 cm^2 in area/get a fester or secondary infection or phlebitis or sterile abscess or wound drainage/seriously affected quality of life<br>[4] Abscess/exfoliativedermatitis/dermal or deep tissue necrosis<br>Duration of symptom: ____________________days
4	☐Itching	[1] Itching at the injection site, resolved spontaneously or resolved within 48 hours after treatment<br>[2] Itching at the injection site, symptom persisted within 48 hours after treatment<br>[3] Affected daily life<br>[4] N.A.<br>Duration of symptom: ____________________days
System adverse events		
5	☐Fever (axillary temperature)	[1] 37.3～＜38.0℃<br>[2] 38.0～＜38.5℃<br>[3] 38.5～＜39.5℃<br>[4] ≥39.5℃， persistent over 3 days<br>Duration of symptom: ____________________days
6	☐Fatigue	[1] Didn't affect daily life<br>[2] Affected daily life<br>[3] Severely affected daily activities and unable to work<br>[4] Went to emergency or needed to be in hospital<br>Duration of symptom: ____________________days
7	☐Diarrhea	[1] Mild or transient, 3-4 times/day, abnormal stool traits or mild diarrhea <1 week<br>[2] Moderate or persistent, 5-7 times/day, abnormal stool traits, or diarrhea >1 week<br>[3] >7 times/day, abnormal stool traits, or

		hemorrhagic diarrhea, orthostatic hypotension, electrolyte imbalance, needed for intravenous infusion >2L<br>[4] Hypotensive shock, needed to be hospitalized<br>Duration of symptom: ____________________days
8	☐muscle pain	[1] Didn’t affect daily life<br>[2] Slightly affected daily life<br>[3] Severe muscle pain and severely affected daily life<br>[4] Went to emergency room or needed to be in hospital<br>Duration of symptom: ____________________days
9	☐Nause	[1] Transient (<24 hours) or intermittent and had normal intake of food<br>[2] Persistent nausea led to decreased food intake (24 to 48 hours)<br>[3] Persistent nausea led to almost no food intake (>48 hours) or intravenous fluid was needed<br>[4] Threatened life (e.g. hypotensive shock)<br>Duration of symptom: ____________________days
10	☐Headache	[1] did’nt affect daily activities and did’nt need treatment<br>[2] Transient, affected daily activities mildly and needed treatment or intervention<br>[3] Affected daily activities severely and needed treatment or intervention<br>[4] Symptom was obstinate, went to emergency room or needed to be in hospital<br>Duration of symptom: ____________________days
11	☐Vomiting	[1] 1~2 times/24 hours but didn’t affected daily activities<br>[2] 3~5 times/24 hours or activity was restricted<br>[3] >6 times within 24 hours or iv. fluid was needed<br>[4] Hypotensive shock led to hospitalization or other means of nutrition was needed<br>Duration of symptom: ____________________days
12	☐Cough	[1] Transient, didn’t need any treatment<br>[2] Persistent, treatment was effective<br>[3] Paroxysmal, treatment was ineffective<br>[4] Went to emergency room or needed to be in hospital<br>Duration of symptom: ____________________days

13	☐Joint pain	[1] Mild pain, didn't affect function<br>[2] Moderate pain/analgesics were needed and/or affected with function but didn't affect daily activities<br>[3] Severe pain, analgesics were needed and/or pain affected daily activities<br>[4] Disabling pain<br>Duration of symptom: ____________________days
14	☐Hypersensitivity	[1] Localized urticaria, didn't need any treatment<br>[2] Localized urticaria, needed treatment/mild angioedema, didn't need treatment<br>[3] Extensive urticaria or angioedema needed treatment, or mild bronchospasm<br>[4] Allergic shock/life-threatening bronchospasm/edema of the larynx<br>Duration of symptom: ____________________days
